# COVID-19 transmission and infection: linkage of COVID-19 Infection Survey, Test and Trace, and Patient Demographics Survey.

**DOI:** 10.23889/ijpds.v7i3.2094

**Published:** 2022-08-25

**Authors:** Leah Maizey, Josie Plachta, Holly Clarke, Sarah Collyer, Sarah Cummins, Gabriela De La Serna, Shelley Gammon, Ben Harries, Elizabeth Pereira, Caroline Youell

**Affiliations:** 1 The Office for National Statistics

## Objectives

Data linkage was conducted between the Office for National Statistics’ Covid Infection Survey (CIS), the Department of Health and Social Care’s Test and Trace (T&T) and NHS’ Personal Demographics Service (PDS) datasets. Linked data was required to provide reliable estimates of rates of COVID-19 transmission and infection used to inform policy regarding the ongoing pandemic.

## Approach

The CIS was created to track infection rates in the UK population. Linking CIS participants to positive tests in T&T helped improve these estimates. Linkage to PDS was required to attach NHS number to these datasets to facilitate further linkages that could also be used to inform Government about the spread of the virus. Multiple approaches were used to link the data. Initially, T&T was linked to itself via a series of strict matchkeys to cluster records belonging to the same individual, to create a person level identifier. Subsequent linkage of CIS-PDS, T&T-PDS and CIS-T&T involved deterministic linkages with matchkeys designed and applied independently. A probabilistic (Fellegi-Sunter scoring) method was used to link CIS-PDS and CIS-T&T. Additional, associative links were created between CIS and T&T records that had matched to the same PDS record but had not matched to each other.

## Results

The accuracy of CIS-PDS and CIS-T&T linkages was high (recall and precision >98%; all 95% lower confidence intervals >93%). A quality assessment of T&T-PDS is underway, as are relevant bias analyses.

## Conclusion

As a result of this linkage, COVID-19 analysts have access to enriched datasets linked to compare previously separated variables, with confidence that the linkage method used was to required quality standards. The linked data has been used to provide crucial evidence to Government on infection and re-infection rates. Subsequent linkages have enabled analysts to explore risk factors associated with different variants of the virus, vaccination status and hospital episodes. Improvements continue to be made.

